# Conjugative type IVb pilus recognizes lipopolysaccharide of recipient cells to initiate PAPI-1 pathogenicity island transfer in *Pseudomonas aeruginosa*

**DOI:** 10.1186/s12866-017-0943-4

**Published:** 2017-02-07

**Authors:** Toan Phuoc Hong, Michelle Q. Carter, Paolo Struffi, Stefano Casonato, Youai Hao, Joseph S. Lam, Stephen Lory, Olivier Jousson

**Affiliations:** 1 Centre for Integrative Biology, University of Trento, 38123 Trento, Italy; 2 Department of Microbiology and Immunobiology, Harvard Medical School, Boston, MA 02115 USA; 3 Department of Molecular and Cellular Biology, University of Guelph, Guelph, ON N1G 2W1 Canada

**Keywords:** *Pseudomonas aeruginosa*, PAPI-1 pathogenicity island, Horizontal gene transfer

## Abstract

**Background:**

*Pseudomonas aeruginosa* pathogenicity island 1 (PAPI-1) is one of the largest genomic islands of this important opportunistic human pathogen. Previous studies have shown that PAPI-1 encodes several putative virulence factors, including a major regulator of biofilm formation and antibiotic-resistance traits. PAPI-1 is horizontally transferable into recipient strains lacking this island via conjugation mediated by the specialized type IV pilus. The PAPI-1 encodes a cluster of ten genes associated with the synthesis and assembly of the type IV pilus. The PAPI-1 acquisition mechanism is currently not well understood.

**Results:**

In this study, we performed a series of conjugation experiments and identified determinants of PAPI-1 acquisition by analyzing transfer efficiency between the donor and a series of mutant recipient strains. Our data show that common polysaccharide antigen (CPA) lipopolysaccharide (LPS), a homopolymer of D-rhamnose, is required for initiating PAPI-1 transfer, suggesting that this structure acts as a receptor for conjugative type IV pilus in recipient strains. These results were substantiated by experimental evidence from PAPI-1 transfer assay experiments, in which outer membrane or LPS preparations from well-defined LPS mutants were added to the transfer mix to assess the role of *P. aeruginosa* LPS in PAPI-1 transfer and *in vitro* binding experiments between pilin fusion protein GST-pilV2’ and immobilized LPS molecules were performed. Our data also showed that *P. aeruginosa* strains that had already acquired a copy of PAPI-1 were unable to import additional copies of the island, and that such strains produced proportionally lower amounts of CPA LPS compared to the strains lacking PAPI-1.

**Conclusions:**

These results suggest that a PAPI-1 exclusion mechanism exists in *P. aeruginosa* that might serve to regulate the avoidance of uncontrolled expansions of the bacterial genome.

**Electronic supplementary material:**

The online version of this article (doi:10.1186/s12866-017-0943-4) contains supplementary material, which is available to authorized users.

## Background

Horizontal gene transfer (HGT) mediated by microorganisms is a major evolutionary mechanism for the acquisition of new functionalities. HGT allows rapid and drastic changes in bacterial genomes, as up to hundreds of new genes can be acquired during a single genetic exchange event. HGT is recognized to play an important role in the evolution of virulence, antibiotic resistance and adaptation to the new environments [1, 2]. The acquisition of virulence genes may radically alter the disease-causing potential of a microorganism. In some instances, acquisition of a single gene or a small cluster of genes encoding critical virulence determinants may be the only genetic difference between avirulent and virulent strains of the same species [3, 4]. Virulence genes are often part of a large block of DNA referred to as genomic islands (GIs). GIs are accessory genomic segments present only in certain bacterial strains; they are often flanked by direct repeats and inserted in the vicinity of tRNA genes. Reversible excision and integration further implicate their potential for inter-bacterial transfer [5]. Those GIs that lead to enhancement of fitness in a host organism are called pathogenicity islands [6]. Integrative and conjugative elements (ICEs), or conjugative transposons, are well-characterized GIs that in many cases have retained mobility [7, 8]. In contrast, a number of GIs appear to belong to the group of ancient ICEs that became fixed in the bacterial chromosome due to degeneration of their conjugative elements by deletion of integration sites or mutations in genes encoding transfer functions [9]. The best-characterized ICEs to date contain specific features associated with conjugative plasmids and bacteriophages; can be transferred horizontally following recognition of the recipient cell by the donor utilizing a conjugative mechanism that, in many instances, is associated with the type IV protein secretion pathway [10]. The recipient cell is recognized by the pilus structure that is part of the type IV secretion apparatus of the donor [11].

*Pseudomonas aeruginosa* is highly adaptable to survive in a wide range of environmental niches; this ability is reflected by its large genomic repertoire. Indeed, the genome database for *P. aeruginosa* strains available to date show that this species possesses a large core genome of ca. 5000 conserved genes and an accessory gene pool of 1000–1500 additional genes; most of the latter are known to be arranged in a limited number of genomic islands [12]. PAPI-1 is one of the largest GIs characterized in *P. aeruginosa* PA14 [13], a highly virulent strain that can infect a broad range of plants, insects, and animals. It is integrated at the *attB* site, juxtaposed to *tRNA*-*lys* genes [14] and consisted of a cluster of 108 genes that encode a number of virulence determinants, whose disruption resulted in the attenuation of the virulence phenotype in several infection models [13]. In addition, PAPI-1 carries several regulatory genes, such as the one that encodes PvrSR/RcsCB two-component system, which controls biofilm formation and dispersal in *P. aeruginosa* strains causing chronic infections in individuals with cystic fibrosis [15]. PAPI-1 island is present in wild-type PA14 strain, whereas, it is not found in PAO1. However, it can easily be transferred from PA14 to PAO1. PAPI-1 transfer has previously been described as a conjugation process mediated by type IVb pilus in co-culture experiments with donor and recipient cells [14, 15]. Type IVb pilus is encoded by a 10-gene cluster in PAPI-1 [15] and is closely related to the genes found in the enterobacterial plasmid R64. Previous studies on conjugal plasmid R64 suggested that the thin pilus, PilV adhesin, was formed by a recombinant mechanism between various cassettes, and a shufflon [16] presumably recognizes a specific structure of the lipopolysaccharide molecules of recipient cells, determining the transfer specificity of the plasmid R64 [17].

The aim of this study was to investigate the mechanism of acquisition of PAPI-1 in *P. aeruginosa*. We performed a series of conjugation experiments on wild-type or mutant donor and recipient strains, and analyzed the determinants that dictate PAPI-1 transfer efficiency. We demonstrated for the first time that the conjugative type IVb pilus of the donor can recognize CPA LPS on the recipient outer membrane, and that this structure is required to initiate the transfer of PAPI-1. Our data also indicate that *P. aeruginosa* strains containing PAPI-1 specify a mechanism to exclude additional copies of PAPI-1 by shutting down the CPA LPS biosynthesis.

## Methods

### Strains, plasmids, and culture conditions

All strains and plasmids used in this study are listed in Additional file 1: Table S1 and Additional file 2: Table S4. *P. aeruginosa* strains and mutants were grown in lysogeny broth (LB, also called Luria-Bertani medium) (Sigma-Aldrich) supplemented with appropriate antibiotics. For selection of *P. aeruginosa* mutants, the antibiotics used were gentamicin and tetracycline, both at a concentration of 75 μg/ml. For maintenance of plasmids in *Escherichia coli*, the medium was supplemented with ampicillin at 100 μg/ml and chloramphenicol at 34 μg/ml. Isopropyl-D-thiopyranoside (IPTG) was added at a final concentration of 0.5 mM to induce GST-pilV2’ expression in the pGEX-2 T plasmid.

### Construction of PA14Δ*TnC2*::Tc^R^ mutant

The mutant PA14Δ*TnC2*::Gm^R^, derived from PA14 transposon insertion mutant library [18] in which the transposon was inserted at nucleotide 1634 of the PAPI-1 gene RL090 (PA14_59200), is proficient in transfer of the PAPI-1 and has been used as a donor in PAPI-1 transfer assay [15]. In some experiments, a donor with tetracycline-resistant (Tc^R^) marker was required since the recipient contained a gentamicin-resistance (Gm^R^) gene, and one would expect to have an equivalent transfer efficiency to the PA14Δ*TnC2*::Gm^R^. The knockout mutant was constructed by using gene replacement vectors as previously described [19]. All primers used for generating the mutant PA14Δ*TnC2*::Tc^R^ are listed in Additional file 2: Table S4. Briefly, a cassette conferring Tc^R^ flanked by two DNA fragments of about 500 bp flanking the PA14*_*59200 gene was cloned in the vector pJET1.2 before subcloning into the vector pEXG2. The recombinant plasmid was conjugated from *E. coli* λpir S17.1 into *P. aeruginosa* [20]. The integrative plasmids were selected on LB agar medium supplemented with gentamicin, tetracycline or irgasan at 25 μg/ml. To resolve merodiploids, a second selection round on LB agar medium with 6% sucrose was performed. Transformants were screened by colony PCR and confirmed by DNA sequencing.

### Screening for PAO1 mutants deficient in PAPI-1 acquisition

A standard PAPI-1 transfer assay via liquid mating was carried out as previously described [15]. Briefly, mutant PA14Δ*TnC2*::Gm^R^ was used as donor and a series of PAO1 mutants, with altered LPS biosynthesis, obtained from a PAO1 transposon mutant library were used as recipients (Additional file 3: Table S2). After overnight growth at 37 °C with shaking at 200 rpm, the donor cells were harvested at an OD_600_ of 0.8 and were mixed with the recipient cells at an OD_600_ of 0.4, spun down and resuspended in 1 ml of fresh LB without antibiotics. The mating mixture was incubated in 15-ml culture tubes, statically at 37 °C for 24 h. The mating mixture was diluted to appropriate dilutions and plated on LB agar medium supplemented with gentamicin and tetracycline at 75 μg/ml to select for transconjugants, and on LB agar plates containing tetracycline at 75 μg/ml to select for recipients. The transfer efficiency was calculated as a ratio of transconjugants to recipients colonies in the mating mixture.

### Outer membrane (OM) preparation

The outer membrane of *P. aeruginosa* was isolated by using sodium lauroylsarkosinate (sarkosyl) as previously described [21]. Briefly, cultures of *P. aeruginosa* were grown overnight at 37 °C with shaking at 200 rpm in LB broth. The pre-inoculum was then diluted 100-fold in fresh LB medium and grown at 37 °C with shaking at 200 rpm to an OD_600_ of 1.0. Cells were harvested and resuspended in 15 ml lysis buffer containing 20 mM Tris-Cl pH 7.5, 100 mM NaCl, 1 mM EDTA, lysozyme 0.5 mg/ml and a complete protease inhibitor cocktail (Roche). The lysate was sonicated and centrifuged at 10,000 × *g*, 10 min, 4 °C to remove cellular debris. The membrane fraction was isolated by ultracentrifugation (200,000 × *g* at 4 °C for 60 min). The pellets containing inner and outer membranes were further fractionated at 100,000 × *g* for 30 min after incubation with 0.2% sarkosyl. The pellet contained outer membranes, which were finally resuspended in Tris-Cl buffer 20 mM pH 7.5 and resolved on 12% sodium dodecyl sulfate (SDS)-polyacrylamide gels and stained with Coomassie blue. The concentration of OM was measured by the standard Lowry method using *DC*™ Protein Assay kit (BIO-RAD) with BSA protein as a standard.

### LPS preparation

For pilin-LPS binding assays, LPS from various *P. aeruginosa* strains and mutants were prepared by using the hot phenol-water extraction protocol from Westphal and Jann (1965) with minor modifications [22]. Briefly, cell suspensions in 100 mM NaCl were first heated to 68 °C before adding an equal volume of hot phenol and stirring vigorously for 2 h at 68 °C. LPS was then fractionated by centrifugation at 12,000 × *g* for 15 min, at 4 °C. LPS in the upper phase was recovered and dialyzed against water to remove residual phenol. LPS extract was further treated with DNase, RNase and proteinase K to eliminate contaminations. LPS extract was finally lyophilized before use. The LPS samples were analyzed by SDS-PAGE and silver staining [23]. Quantitation of LPS was performed by using a Kdo assay as described previously [24].

### PAPI-1 transfer inhibition assay

OM and LPS preparations at various concentrations (0; 0.5; 1.0; 2.0; 5.0; 10.0 and 15.0 μg) were added to a standard mating assay based on plasmid conjugal transfer [25], between the donor PA14Δ*TnC2*::Gm^R^ and the recipient PAO1::Tc^R^. A mating mixture without the addition of OM or LPS was also included as a negative control for this experiment. The transfer inhibition index was calculated by dividing the transfer efficiency observed with the addition of OM or LPS to that of the control.

### Western blotting for LPS samples

LPS samples prepared by the Hitchcock and Brown method [26], were used for SDS-PAGE, silver staining and Western immunoblotting, following protocols that had been described previously [22]. Briefly, 3 μl of LPS samples was loaded into the 12% SDS-PAGE and run at 80 V for 120 min. LPS was electrophoretically transferred onto nitrocellulose membrane at 180 mA for 50 min. The membrane was then blocked with 5% skim milk for 30 min at room temperature. The membranes were washed in PBS for 3 × 10 min; the primary antibodies monoclonal antibodies (MAb) against different regions of the LPS structure, including MF15-4 (O-specific antigen specific), N1F10 (CPA specific), 5c101 (outer core specific), and 5c-7-4 (inner core specific), were added and incubated overnight at room temperature. Following a 3 × 10 min PBS wash, secondary antibodies were added for an additional hour. The membrane was washed for 2 × 10 min in PBS, 10 min in buffer A before developed by BCIP/NBT detection kit.

### Expression and purification of GST-pilV2’ fusion protein

The C-terminal region of pilV2 gene encoding 97 residues was amplified with the primers listed in Additional file 2: Table S4 and cloned into pJET 1.2/Blunt. The insert was then subcloned into the expression vector pGEX-2 T (Life Technologies) and transformed into *E. coli* BL21. *E. coli* BL21 containing pGEX-2 T-pilV2’ was grown to an OD_600_ of 0.6 at 37 °C with shaking at 200 rpm, before adding IPTG to induce expression of GST-pilV2’ protein. The culture was incubated for additional 3 h. The cells were collected by centrifugation at 12,000 × *g*, at 4 °C for 20 min. The GST-pilV2’ fusion protein was then purified by using glutathione Sepharose 4B (GE Healthcare), as previously described [27].

### Microtiter plate binding assay

Binding of LPS to GST-pilV2’ fusion was quantified by a modified enzyme-linked immunosorbent assay (ELISA) essentially as described previously [28]. Microtiter plates (Corning) were coated with 10 μg/ml LPS from PAO1 and PAO1 with PAPI-1 suspended in PBS (0.137 M NaCl, 0.005 M KCl, 0.009 M Na_2_HPO_4_, and 0.001 M KH_2_PO_4_ (pH 7.4)) containing 0.05% Tween 20 (v/v, PBST). The plates were then washed with PBST and blocked with 3% BSA. GST-pilV2’ fusion was added to the wells coated with LPS and incubated for 2 h at room temperature. After three washes with PBST, mouse anti-GST antibody was added and incubated for 1.5 h following three washes with PBST, HRP-labeled anti-mouse Ig (Sigma-Aldrich) was added for 1 h, followed by three additional washes. A solution of 3,3′,5,5′-tetramethylbenzidine (Thermo Scientific) was used for color development at A_450_. LPS from *Salmonella enterica* was used as a negative control.

## Results

### PAO1 derivatives defective in CPA LPS biosynthesis are deficient in the acquisition of PAPI-1

It has been known that PAPI-1 island is transferable from one to another *P.aeruginosa* strain lacking it by conjugation mechanism via type IVb pilus. PAPI-1 island carries a 10-gene cluster (*pilL2*, −*N2*, −*O2*, −*P2*, −*Q2*, −*R2*, −*S2*, −*T2*, −*V2*, and -*M2*) encoding structural and assembly accessory proteins of type IVb pilus such as PilS2, the major subunit of the pilus filament and PilV2, the minor subunit of the pilus filament. Insertion of the transposon into any of 10 *pil* genes led to a significant loss of PAPI-1 transfer efficiency [15]. Here, we confirmed that the transfer efficiency values of PA14ΔpilV2 and PA14ΔpilS2 are <10^−7^, which is significantly lower than the positive control mutant PA14Δ*TnC2*::Gm^R^, previously been proposed as a proficient donor in PAPI-1 transfer [15].

**Fig. 1:**
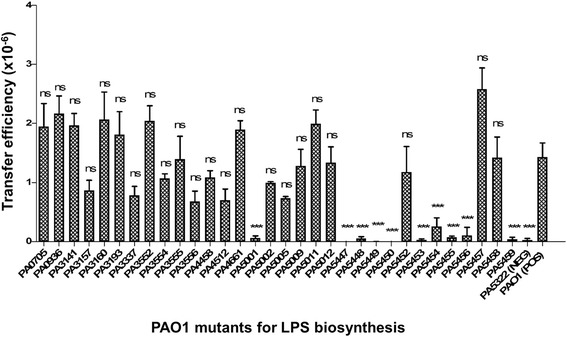
PAPI-1 transfer efficiency into various PAO1 mutants for LPS biosynthesis as recipient strains. Positive control (POS): PAO1::Tc^R^ and Negative control (NEG): PA5322 (or PAO1Δ*algC*). Results were shown as mean ± SD for three independent replicates. Statistical significance was assessed by One-way ANOVA compared to the positive control (ns: no significant difference; and *** *p* < 0.001)

Since in conjugal plasmid system the donor pilus is known to interact with specific components of LPS on the recipient membrane to initiate the transfer [29], we examined the effects of using various PAO1 mutants for LPS biosynthesis as recipients on PAPI-1 transfer assay (Fig. 1). We first checked the transfer efficiency of PAPI-1 from PA14Δ*TnC2*::Gm^R^ into the mutant PAO1∆*algC*, in which *algC* gene was disrupted by transposon insertion at nucleotide 628 [30]. The gene *algC* encodes a phosphoglucomutase, which is required for the synthesis of a complete LPS structure [31]. The mutant PAO1Δ*algC* thus produces a truncated LPS core and is devoid of O-antigen. When the transfer assay was carried out between the donor PA14Δ*TnC2*::Gm^R^ and PAO1Δ*algC*::Tc^R^ (PA5322), the transfer efficiency was reduced by three orders of magnitude compared to that of wild-type PAO1 (Fig. 1). This suggests that a complete LPS structure plays an important role in PAPI-1 acquisition. We therefore decided to screen a panel of 32 PAO1 mutants for LPS biosynthesis (Additional file 3: Table S2) [32] using them as recipients in the PAPI-1 transfer assay. These mutants were obtained from the PAO1 transposon mutant library, which contain Tc^R^ [30].

The results showed that transfer efficiency of PAPI-1 into 10 PAO1 mutants including PA5001; PA5447 (*wbpZ*); PA5448 (*wbpY*); PA5449 (*wbpX*); PA5450 (*wzt*); PA5453 (*gmd*); PA5454 (*rmd*); PA5455; PA5456 and PA5459 was significantly decreased by two to three orders of magnitude, compared to the PAO1 control. All these genes, except PA5001, were predicted as members of two operons [33]. The first one is at coordinates 6135968–6144991 (8 genes) and the other one at 6145399–6151151 (5 genes). Interestingly, these genes are known to be involved in Common Polysaccharide Antigen (CPA) biosynthesis (Additional file 4: Table S3). The genes *algC, wbpZ, wbpY, wbpX, wzt, wbpM, gmd, rmd* encode enzymes involved in CPA biosynthesis [32], as shown in Additional file 5: Figure S1, while the genes *PA5455, PA5456, PA5459* encode transferase enzymes [34]. The CPA or A-band LPS has been shown to contain a tri-saccharide repeating unit: [→3)-α-D-Rha-(1→3)-α- D-Rha-(1→2)-α- D-Rha-(1→]_n_, so-called D-rhamnose homopolymer or rhamnan structure. This structure has been characterized by different authors with good agreement between different studies [35, 36]. Our data support the idea that the CPA could act as a receptor for conjugative type IV pilus as an initial step of PAPI-1 transfer. Noteworthy, the *rmd* mutant showed a slightly higher efficiency compared to the negative control which can be explained by the complementary effect of *gmd* gene. The RMD enzyme encoded by *rmd* gene catalyzes the final step in the GDP-D-Rha biosynthesis pathway. The disruption of *rmd* gene in *P. aeruginosa* chromosome impairs CPA synthesis [37], while *gmd* is partially capable of catalyzing the same reaction as RMD enzyme in vitro [38]. Therefore, *gmd* could complement CPA biosynthesis in the *rmd* mutant. We also observed that the transfer efficiency of the *wbpW* mutant (PA5452) was comparable to the positive control. This is not surprising since it has been recently found that *PslB* gene is able to substitute *wbpW* to promote CPA production in a *wbpW* mutant [39] at a low abundance [37]. Here, to confirm if *pslB* plays a role in PAPI-1 transfer, we carried out transfer assay between PA14ΔTnC2 donor and those PAO1 mutants. Our result showed that the transfer efficiency of PAO1Δ*pslB* is comparable to PAO1Δ*wbpW*, while it is significantly decreased by about 20-fold as compared to that of PAO1Δ*wbpW/ pslB* (Additional file 6: Figure S2 and Additional file 7: Table S5). The genes *PA5455, PA5456, PA5459* are located in a five-gene operon (PA5455–PA5459). The enzymes *pa5455* and *pa5456* were recently found to be essential for CPA biosynthesis [34]. Mutants in these genes are devoid of CPA, and show a reduced PAPI-1 transfer efficiency (Fig. 1). To the contrary, enzymes *pa5457* and *pa5458* are not essential for biosynthesis of a CPA structure [34], and mutants in these genes showed comparable transfer efficiency as the wild-type strain. Noticeably, the mutant PA5459 exhibited significantly reduced PAPI-1 transfer efficiency even though it is known to produce observable changes in CPA phenotypes. Mutants involved in core LPS synthesis such as *waaC, waaF and waaP*, did not show a significant decrease in PAPI-1 transfer. These results suggest that the CPA is the main LPS structure driving the contact and interaction between donor and recipient in *P. aeruginosa*.

### Addition of OM and CPA LPS preparations inhibits PAPI-1 transfer

**Fig. 2:**
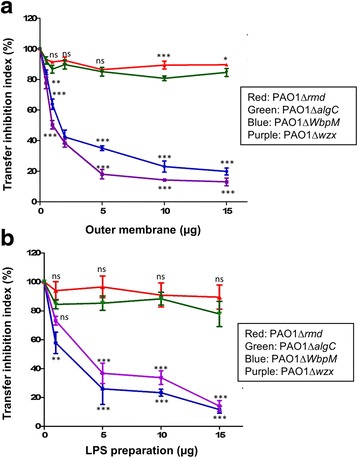
Effect on PAPI-1 transfer efficiency of the addition of OM (a) and LPS (b) preparations from PAO1 mutants lacking either CPA or OSA. *Red*, PAO1Δ*rmd* (A-,B+); green, PAO1Δ*algC* (A-, B+); *blue*, PAO1Δ*wbpM* (A+, B-); *purple*, PAO1Δ*wzx* (A+,B-). Results were shown as mean ± SD for three independent experiments. Two-way ANOVA followed by Bonferroni post-test was used to analyze the data. All samples were compared to OM (**a**) or LPS (**b**) derived from PAO1- cells (ns: no significant difference; *, *p* < 0.05; **, *p* < 0.01; ***, *p* < 0.001)

We postulated that either OM fractions or CPA LPS, the putative receptor for conjugative pilus, could compete with recipient cells for binding to the conjugative pilus; hence the use of these *P. aeruginosa* cell envelope components might block the transfer of PAPI-1 to the recipient. We prepared OM and LPS from isogenic mutants of *P. aeruginosa* PAO1; two of the mutants were defective in CPA LPS biosynthesis (Δ*rmd* and Δ*algC*) and two other mutants were defective in OSA LPS (Δ*wbpM* and Δ*wzx*). These preparations were added to the standard mixture of PAPI-1 transfer assay at different amount from 0 to 15 μg. OM and LPS preparations from OSA-mutants (Δ*wbpM* and Δ*wzx*) strongly inhibited transfer even at low concentrations (<5 μg) and reached a maximum inhibition of about 80% (Fig. 2; Additional file 8: Table S7 and Additional file 9: Table S8). In contrast, OM preparation and LPS from CPA-mutants (Δ*rmd* and Δ*algC*) did not significantly affect the efficiency of PAPI-1 transfer. These results strongly support our hypothesis that CPA LPS in *P. aeruginosa* acts as a specific receptor for the IVb pilus, and is required to initiate the transfer of PAPI-1.

### Recipient strains carrying PAPI-1 show a significant reduction in its ability to acquire additional copies of the island

**Fig. 3:**
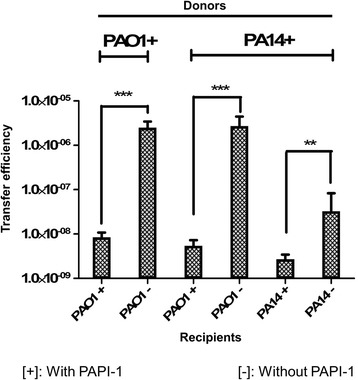
Transfer efficiency of multiple PAPI-1 copies into recipient strains. Marks [+] or [−] stand for strains with or without PAPI-1, respectively. After acquisition of PAPI-1, PAO1 becomes a stable donor which can transfer PAPI-1 to another recipient and decreases its ability of receiving additional copies of PAPI-1. Results are presented as mean ± SD for three independent experiments. Statistical significance was calculated by the unpaired t-test (** *p* < 0.01, and *** *p* < 0.001)

To address the question of whether *P. aeruginosa* strains already carrying PAPI-1 could acquire additional copies of this island, we carried out transfer assays using the donor PA14Δ*TnC2*::Gm^R^ or PAO1*Bla6TnC2*::Gm^R^ and the recipients with PAPI-1 (PA14Δ*TnC2*::Tc^R^ or PAO1*Bla6TnC2*::Tc^R^) or without PAPI-1 (PA14Δ*soj* and PAO1). The transconjugants carrying more than one copy of PAPI-1 were selected with double antibiotic-resistant markers (Gm and Tc). Herein, we designated [+] and [−] for strains with and without PAPI-1, respectively. The transfer efficiency of PA14+ to PA14- is significantly lower than to that of the control PAO1- (Fig. 3; Additional file 10: Table S6). This observation suggested that the transfer efficiency of PAPI-1 is strain-dependent. Interestingly, our data show that PAO1 strain carrying PAPI-1 can act as a donor of the island, transferring it to the control recipient (PAO1-) at an efficiency level comparable to the PA14+ donor. These results also demonstrated the ability of the recipient strains to acquire more than one copy of PAPI-1, even if this occurs at a much lower efficiency as compared to the control, with a decrease of one and three orders of magnitude for PA14+ to PA14+ and PAO1+ to PAO1+ transfers, respectively. Our data thus indicate that *P. aeruginosa* strains which already acquired PAPI-1 showed a significant decrease in their ability to receive additional copies of the island. This implies that *P. aeruginosa* strains carrying PAPI-1 specify a mechanism to exclude the acquisition of additional copies of the island.

### The acquisition of PAPI-1 activates a surface exclusion mechanism

**Fig. 4:**
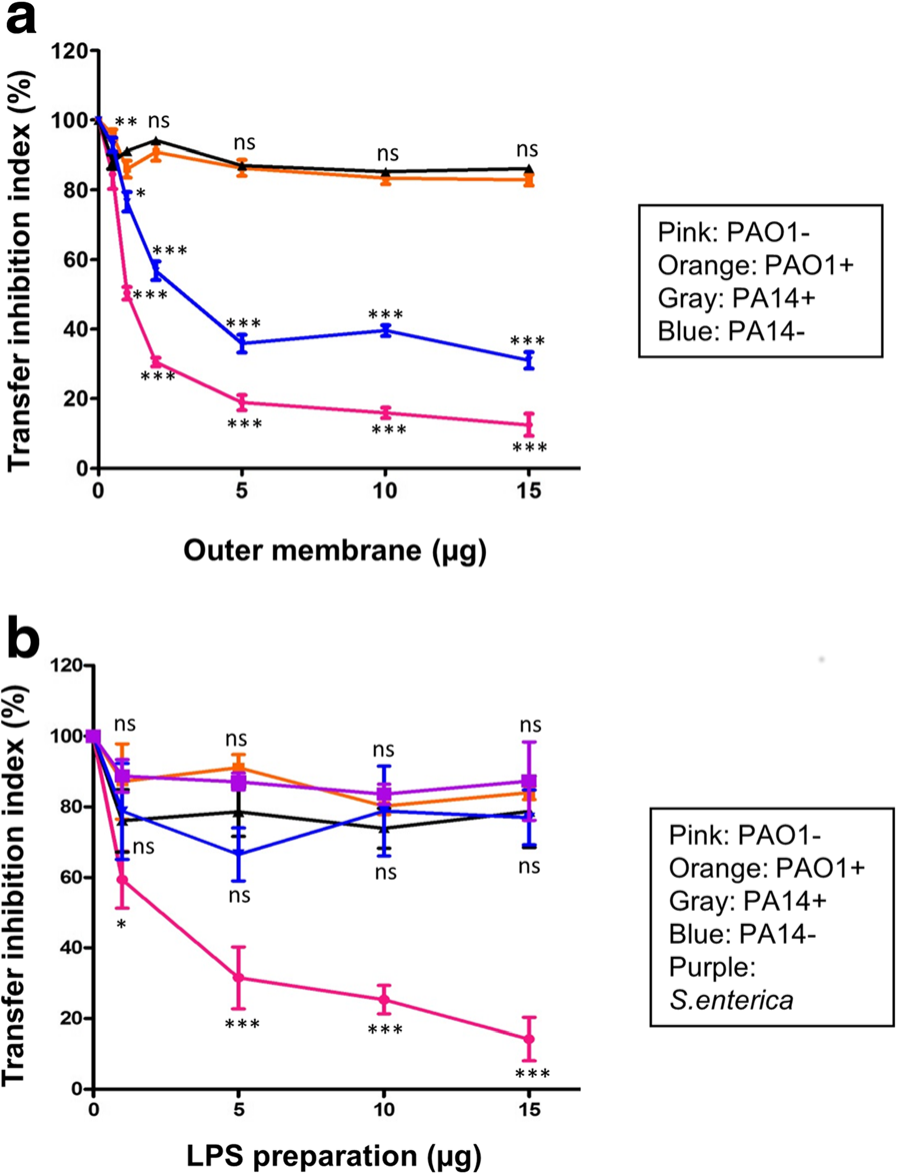
Effect on PAPI-1 transfer of the addition of OM (**a**) and LPS (**b**) preparations derived from strains with (+) or without (−) PAPI-1 island. *Pink*, PAO1-; *orange*, PAO1+; *black*, PA14+; *blue*, PA14-. *Purple* data point on (**b**) are LPS from *Salmonella enterica* used as negative control. Results were shown as mean ± SD for three independent experiments. Two-way ANOVA followed by Bonferroni post-test was used to analyze the data. All samples were compared to OM (**a**) or LPS (**b**) derived from PAO1- cells (ns: no significant difference; *, *P* < 0.05; **, *P* < 0.01; ***, *P* < 0.001)

We hypothesized that after acquisition of PAPI-1, recipient *P. aeruginosa* strains would modify their surface to avoid further contact and subsequent transfer from the donor cells. To verify if the presence of PAPI-1 in the cell genome can affect the structure of its OM and LPS, we performed the standard transfer assay with the addition of OM and LPS, prepared from strains with and without PAPI-1. The addition of OM preparations derived from strains with PAPI-1 did not impact the transfer efficiency, when compared to the OM prepared from strains without the pathogenicity island (Fig. 4a). The effect of LPS preparations (Fig. 4b) is similar as that of OMs for strain PAO1, but not for PA14. Indeed, the addition of LPS from PA14- did not induce any significant variation in transfer efficiency. Experimental data were also shown in Additional file 8: Table S7 and Additional file 9: Table S8.

### Reduction of CPA LPS production plays a role in the surface exclusion mechanism of PAPI-1

**Fig. 5:**
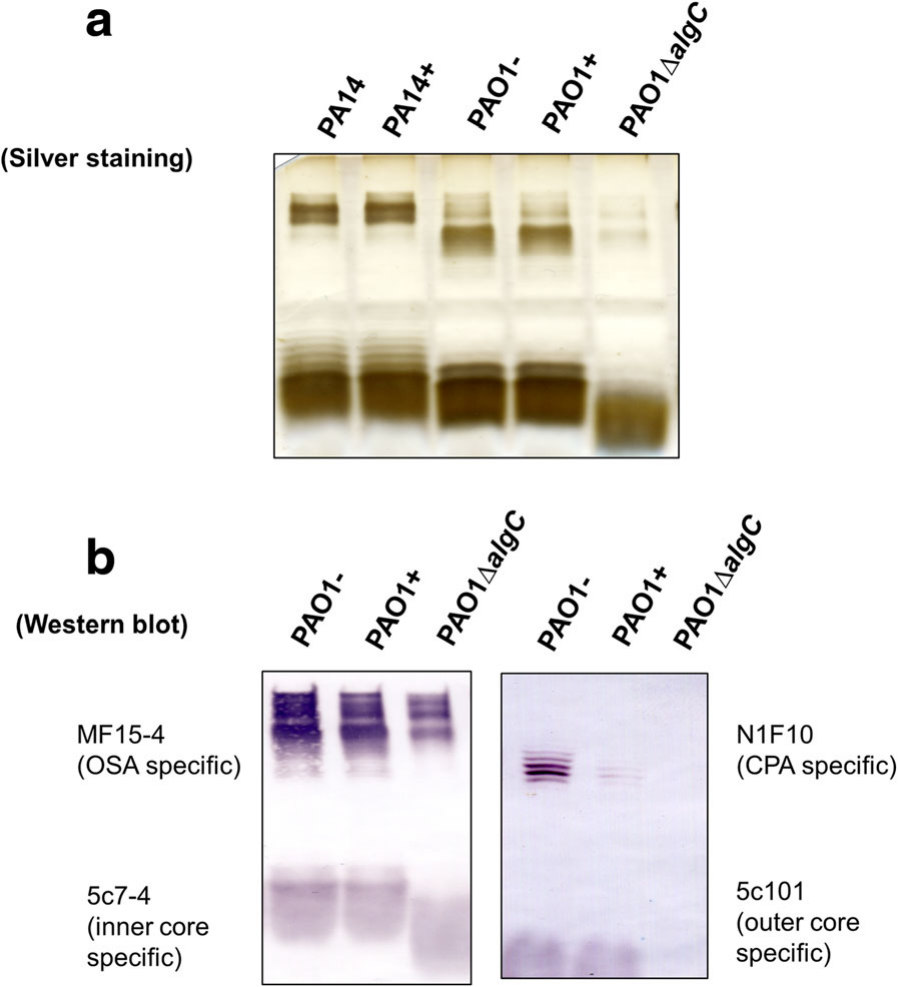
Analysis of LPS preparations from PAO1 and PA14 strains with (+) or without (−) PAPI-1. **a** LPS silver staining. **b** Western blot with a combination of antibodies specifically recognizing A-band (N1F10), B-band (MF15-4), outer core (5c101) and inner core (5c7-4)

**Fig. 6:**
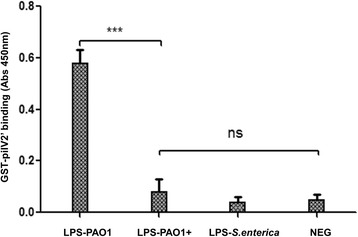
Binding of GST-pilV2’ to various LPS preparations in an enzyme-linked immunosorbent *assay.* LPS from *Salmonella enterica* and sample without LPS used as negative control. LPS derived from PAO1 carrying PAPI-1 showed the loss of binding capacity to GST-pilV2’. Results were presented as mean ± SD for three independent experiments. Statistical significance was assessed by One-way ANOVA (*** *p* < 0.001; ns: no significance, *P* > 0.05)

To better understand the putative mechanism utilized by *P. aeruginosa* to exclude additional copies of PAPI-1, we first considered potential alterations in surface exclusion. We therefore asked whether the strains containing PAPI-1 exhibits altered LPS structure, which would result in the reduction of the donor’s pilus ability to bind to its receptor. Results from silver-stained SDS-PAGE of LPS did not show any discernible differences between the LPS banding patterns in a given strain with or without PAPI-1 (Fig. 5a). We then performed Western immunoblotting using a combination of monoclonal antibodies (MAb) that recognized distinct regions within *P. aeruginosa* LPSs, including MF15-4 (serotype O5 OSA-specific) [40, 41]; N1F10 (CPA-specific) [40, 42]; 5c-7-4 (inner core-specific) [40, 43]; 5c-101 (outer cores-pecific) [40, 43]. This could not be done for PA14 strains because the corresponding set of MAb are not available. We observed that there are no differences between different parts of LPS, with the notable exception of the CPA component from PAO1+ cells, which is significantly reduced compared to PAO1- cells (Fig. 5b). This result could be explained assuming that, after acquiring a PAPI-1 island, PAO1 downregulates the expression of genes involved in the biosynthesis of CPA LPS, leading to a reduced ability to bind the conjugative pilus. To test whether the PAO1+ strain had lost the ability to recognize the donor conjugative pilus, we over-expressed and purified a GST-pilV2’ fusion protein and performed ELISA to compare the *in vitro* binding capacity of pilV2’ to LPS derived from PAO1+ and PAO1- strains. PilV2’ is a small pilin protein constituting the type IVb pilus [15] showing significant similarity to adhesins PilVB and PilVA’ of plasmid R64, with 40% identity in amino acid sequence (Additional file 11: Figure S3). C-terminal variable segments of R64 pilV adhesins were previously shown to interact in vitro with LPS of recipient cells [17]. Therefore, we engineered a glutathione transferase (GST) fusion protein carrying at its C-terminus a 97-amino acid C-terminal segment of PilV2, so-called GST-pilV2’ fusion. This is analogous to the construct used to analyze the R64 pilin interactions with its receptor. This GST-PilV2’ fusion was then tested for binding to various LPS preparations. The binding capacity of GST-PilV2’ to LPS from PAO1+ was significantly decreased compare to binding with LPS from PAO1- (Fig. 6). These observations support the hypothesis that the acquisition of PAPI-1 would result in the modification of PAO1’s LPS, which in turn might cause the loss of the ability to interact with the pilus from donor strains.

## Discussion

It is well known that horizontal gene transfer plays an important role in driving bacterial evolution and adaptation to survive in various environments. *P. aeruginosa* genome has a mosaic structure composed of a variable number of horizontally-acquired accessory regions containing up to hundreds of genes [12]. PAPI-1 is the largest genomic island in *P. aeruginosa*, and has previously been shown to be transferable to recipients lacking it through direct cell-to-cell interaction and by a conjugation mechanism [15].

Here, we demonstrate, for the first time, that *P. aeruginosa* PAO1 derivatives defective in CPA biosynthesis are significantly affected in their ability to acquire PAPI-1, and that addition of outer membrane or CPA LPS preparations from wild-type PAO1 significantly inhibits PAPI-1 transfer. These data suggest that CPA LPS on the recipient membrane plays a significant role during the conjugation and transfer of PAPI-1. LPS has been shown to be the receptor for transfer of different plasmids and bacteriophages. For example, the R64 plasmid transfer is mediated by the recognition of specific parts of the LPS core. Specifically, the GlcNAc(α1-2)Glc or Glc(α1-2)Gal structures, are recognized by PilVB′ and PilVC′ adhesins, respectively [29]. Moreover, LPS molecules have been found as receptors for many bacteriophages [44]. The D-rhamnose-containing CPA-LPS has been shown to be a receptor for bacteriophage A7, and the phage is capable of hydrolyzing the CPA-LPS to expose core-lipid A [45]. Our data strongly suggests that the conjugative pilus interacts with CPA-LPS on the surface of the recipients in order to initiate the transfer of PAPI-1.

Our work suggests that the PAPI-1 transfer occurs toward another *P. aeruginosa* strain producing CPA LPS or homopolymer of D-rhamnose (D-Rha) structure (from PA14 toward PAO1). CPA is one of the two types of O-polysaccharides (OPS) produced by the majority of *P. aeruginosa* strains [32]. Homologous CPA biosynthesis genes have also been found in the genome of *P. fluorescens* pfO-1 [46], but there is no report about LPS structure of this strain to date. On the other hand, the same structure of D-Rha repeating units [→3) D-Rha(α1 → 3) D-Rha(α1 → 2) D-Rha(α1→)] has also been found in the LPS of several other bacterial species including the plant pathogen *Xanthomonas campestris* pv. *phaseoli* var. *fuscans* [47], the opportunistic human pathogen *Stenotrophomonas maltophilia* serotype O7 [48], *P. syringae* strains [49] and a *Burkholderia cepacia* strain [50]. The presence of the same LPS structure in different bacterial species raises the possibility that the PAPI-1 island may be transferable among those species. Future studies need to address this intriguing possibility that would enhance our knowledge about the mobilization of genetic elements in bacterial communities in the human body and in the environment.

We also demonstrated that following acquisition of the PAPI-1island, the recipients can become stable donors for transfer of the island to other recipient cells. Remarkably, PAPI-1 acquisition was stable after several generations and in fact during evolution PAPI-1 is partially or entirely retained in some clinical isolates [13]. This would suggest that the acquisition of PAPI-1 would probably be beneficial to the host though it is not well understood in which manner. However, since the size of PAPI-1 is quite large, about 100 kb, it is likely to cause inconvenience to the cells due to metabolic cost and genome expansion. Similarly to conjugative plasmid, the recipients may respond to a number of incoming ICEs [51].

Herein, we demonstrated that the recipient which already acquired PAPI-1 island were able to receive more copies though transfer efficiency was significantly decreased. At present, we have not collected data to define how many copies of PAPI-1 that recipient would have the capacity to receive from the donor strain and how long it might maintain it; however, the occurrence of multiple copies of PAPI-1 at the attB site as a tandem array was previously reported [14]. The pathogenicity islands in *P.aeruginosa*, PAPI-1 and PAPI-2, are known to be inserted and excised at the specific att sites located in the two tRNA^Lys^ genes, which were identified as “hot spots” for insertion and excision of large genetic elements in several *P. aeruginosa* strains [14]. For instance, the large plasmid pKLK106 in *P. aeruginosa* clone K was able to recombine sequentially with either of the two tRNA^Lys^ genes PA4541.1 and PA0976.1 to rearrange the genomes of sequential K isolates from the airway of a CF patient [52]. In *P. aeruginosa* clone C, the plasmid pKLC102 reversibly integrated only into PA4541.1 but not into PA0976.1, which was occupied by a 23-kb PAGI-4 island [53]. In *Vibrio cholerae*, it was reported that tandem arrays of SXT and R391 elements occurred after their transfer, and this arrangement was observed to be stably maintained for many generations [54]. These suggested that the *attB* site in a the genome of a recipient can be used as a platform to build composite GIs by sequentially acquired independent genetic elements to form a superintegron [55]. Finally, the integration of PAPI-1 into the site for PAPI-2 means that this *attB* site is conserved and remains intact at the borders of the composite element. This feature could be used for acquiring multiple ICEs in *P. aeruginosa*. Harboring at least two ‘’hot spots” for integration of genetic elements, this bacterium is likely to employ an exclusion system to avoid the expansion of its genome and metabolism. This activity has been well documented for conjugative plasmids, but there is not much evidence of this for ICEs. After acquiring a genetic element, the bacteria can modify their cell surfaces or express specific factors to ignore or cleave incoming elements, which are classified in different barrier levels. The bacteria might possess and activate one or some of them to maintain a stable state, and this would affect the efficiency of the transfer after acquiring these elements. In this study, the exclusion activity of PA14 and PAO1 with PAPI-1 were successfully addressed. Exclusion Index (EI) was calculated as the transfer efficiency of PAPI-1 to the recipient lacking this element divided by that to a recipient already carrying the same element [51]. We found that the EI for mating between PAO1+ donor and PAO1+ recipient was about 298. The observed EI values were comparable to those of the SXT family of ICEs [56], but about two-fold lower than that reported for the virulence plasmid pVAPA1037 [57], six-fold lower than for the plasmid R27-mediated entry exclusion [58], and twenty-fold lower than for the highly promiscuous plasmid RP4, which has EI values ranging from 10^3^ to 10^4^ [59]. Moreover, we found that addition of LPS preparations of PAO1- significantly inhibits transfer efficiency of PAPI-1, while addition of LPS preparations of PAO1+ has no significant effect on transfer efficiency of PAPI-1. When we examined the LPS of PAO1 strains, we found that upon acquisition of PAPI-1, PAO1+ reduces the amount of CPA, and this presumably adversely affects the tightness of the contact between donor and recipient cells. These data indicate that reduction of CPA LPS after acquisition of PAP1-1 serves as an exclusion mechanism for acquisition of multiple copies of the PAPI-1 island. It is worth noting that PA14 strain contains a SNP in the CPA biosynthesis gene *wbpX* and naturally does not produce CPA [60]. In fact, in accordance with our proposed role of the reduction of CPA as an exclusion mechanism, we found that the EI value for mating between PA14 donor and PA14+ recipient was 12, which is an order of magnitude lower than that measured for PAO1. Unlike the LPS preparation of PAO1-, the LPS from PA14- did not induce any significant variation in transfer efficiency. Interestingly, the outer membrane preparations of PA14- showed some inhibitory effect to PAPI-1 transfer efficiency, while that of PA14+ did not. This may suggest that in addition to CPA, there might be other exclusion mechanisms present in the outer membrane of this strain.

To date, at least two exclusion mechanisms for plasmid or ICEs acquisition are known. One is the entry exclusion (Eex) mediated by inner membrane proteins. This mechanism is able to inhibit DNA entry after a stable mating pair has been established [56, 59, 61]. The other mechanism occurs via surface exclusion, inhibiting formation of a stable mating pair. An example is TraT, which is an outer membrane protein encoded by the F plasmid. This protein can impede the interaction between the pilus tip and OmpA receptor in *E. coli* [61–63].

More work is required to elucidate the molecular basis for the PAPI-1 exclusion mechanism. Considering that PAPI-1 lacks any identifiable homologues of genes involved in LPS biosynthesis and modification (data not shown), the factors causing a reduction in the amount of CPA LPS may be conceivably located in the core genome of *P. aeruginosa*. PAPI-1 could therefore specify regulatory functions controlling the expression of the enzymes for LPS biosynthesis. Our study provides new insights on the horizontal acquisition and exclusion of genomic islands, which may lead to future development of new strategies to limit the spread of virulence or resistance functions in *P. aeruginosa* populations.

## Conclusions

Horizontal gene transfer (HGT) represents a major evolutionary mechanism for the acquisition of new phenotypes by microorganisms. HGT plays a particularly important role in the evolution of virulence and antibiotic resistance as it allows acquisition of genes that can alter the pathogenic potential of a bacterial strain. To our knowledge, the mechanism of HGT has never been experimentally investigated in *P. aeruginosa*. This may be partly due to the fact that the mobility of these elements is frequently lost because of evolutionary decay. The significance of this work is in our ability to experimentally test the molecular mechanisms of acquisition of the genomic island PAPI-1 by HGT. Indeed, we provided evidence that conjugative type IVb pilus recognizes CPA lipopolysaccharide of recipient cells to initiate PAPI-1 pathogenicity island transfer in *P. aeruginosa.* We also report that the bacterium produces less CPA after acquiring PAPI-1, as a mechanism to exclude the acquisition of additional copies of PAPI-1. New insights about PAPI-1 mobility and its dissemination by HGT could be applicable to other systems where experimental validation of transmission models has not yet been attained.

## Additional files

Additional file 1: Table S1. Strains and plasmids used in this study. (DOC 56 kb)
Additional file 2: Table S4. Primers used in this study. (DOC 45 kb)
Additional file 3: Table S2. List of PAO1 mutants for lipopolysaccharide biosynthesis. (DOC 67 kb)
Additional file 4: Table S3. Genes involved or potentially involved in Common Polysaccharide Antigen (A-band LPS) biosynthesis. This table was reproduced by King *et al.*, 2009. (DOC 69 kb)
Additional file 5: Figure S1. The GDP-D-rhamnose (CPA monomer) biosynthesis pathway. All the enzymes are encoded by a gene cluster, except the second enzyme phosphomannomutase encoded by algC gene in the alginate locus. D-fructose-6-phosphate; 1, Pi, phosphate; 2, mannose-6-Pi; 3, GDP-D-Man; 4, α-mannose-1-Pi; 5, GDP-4-keto-D-Rha; 6. This figure was modified from King et al., 2009 [32]. (TIF 572 kb)
Additional file 6: Figure S2. Role of pslB gene in PAPI-1 transfer. Results were shown as mean ± SD for three independent replicates. Statistical significance was analyzed by One-way ANOVA compared to the positive control (ns: no significant difference; and *** *p* < 0.001). (TIF 217 kb)
Additional file 7: Table S5. Transfer efficiency of different strains and mutants. (DOCX 19 kb)
Additional file 8: Table S7. PAPI-1 transfer inhibition following addition of OMs. (DOCX 24 kb)
Additional file 9:Table S8. PAPI-1 transfer inhibition following addition of LPSs. (DOCX 22 kb)
Additional file 10: Table S6. Transfer efficiency of different combinations of donors and recipients with or without PAPI-1. (DOCX 14 kb)
Additional file 11: Figure S3. Homologues of P.aeruginosa pilV2 sequence and pilV adhesins of plasmid R64 of *Salmonella enterica* serovar Typhimurium LT2 (Multiple Sequence Alignment, Clustal Omega software, EMBL-EBI). (TIF 2355 kb)
