# Supplementary material for: Conjugative type IVb pilus recognizes lipopolysaccharide of recipient cells to initiate PAPI-1 pathogenicity island transfer in Pseudomonas aeruginosa
Source: BMC Microbiol. 2017 Feb 7;17:31. doi: 10.1186/s12866-017-0943-4 (PMC5297154; doi:10.1186/s12866-017-0943-4)
Supplement: Additional file 1: Table S1. — Strains and plasmids used in this study. (DOC 56 kb) [file 12866_2017_943_MOESM1_ESM.doc]

Table S1. Strains and plasmids used in this study

| Strains or plasmids | Antibiotic resistance*^a^* | | Description | Source reference, or accession no. |
| --- | --- | --- | --- | --- |
| *E. coli strains* |  | |  |  |
| *E. coli* SM10 | None | | Host strain for plasmids pEXG2, mini-CTX, and their derivatives | Lory’s lab collection |
| *E. coli* λpir S17.1 | None | | Transfer pEXG2 plasmid into *P. aeruginosa* by conjugation | [1] |
| *P. aeruginosa strains* | |  | |  |
| PA14 | None | | Burn isolate | [2] |
| PA14∆*soj*  (or PA14 -) | Gm^R^ | | Deletion mutant of PAPI-1 *soj* in strain PA14, which does not carry PAPI-1 island | [2] |
| PA14∆*TnC2*::Gm^R^  (or PA14+) | Gm^R^ | | Strain PA14 with a transposon MAR2×T7 inserted at nucleotide 1634 of PAPI-1 gene RL090 (PA14_59200) | [3] |
| PA14∆*TnC2*::Tc^R^ (PA14+) | Tc^R^ | | Deletion of the PA14_59200 gene in strain PA14 by insertion of tetracycline resistant gene in the middle | This study |
| PAO1 (or PAO1 -) | Tc^R^ | | PAO1 with Tet gene inserted at the CTX phage *att* site on the chromosome | Lory’s lab collection |
| PAO1*Bla6* | Cb^R^ | | PAO1 with genes *bla* and *lacZ* inserted at the CTX phage *att* site on the chromosome | [2] |
| PAO1*Bla6TnC2*::Gm^R^ (PAO1+) | Gm^R^  Cb^R^ | | Transconjugant of the mating between PA14∆TnC2 (Gm^R^) and PAO1Bla6 | This study |
| PAO1*Bla6TnC2*::Tc^R^ (PAO1+) | Gm^R^  Cb^R^ | | Transconjugant of the mating between PA14∆TnC2::Tc^R^ and PAO1Bla6 | This study |
| Plasmids |  | |  |  |
| pEXG2 | Gm^R^ | | Gene replacement vector for constructing deletion or insertion mutants of *P. aeruginosa* | [4] |
| pJET1.2 | Amp^R^ | | Plasmid used for DNA blunt cloning | Thermo Scientific |
| pGEX-2T | Amp^R^ | | Expression vector for GST-pilV2’ | GE Healthcare |

[↵](http://jb.asm.org/content/192/13/3249/T1.expansion.html#xref-fn-5-1)a Amp^r^, ampicillin resistance; Gm^r^, gentamicin resistance; Cb^r^, carbenicillin resistance; Tc^r^, tetracycline resistance.

**References**

1. Simon R, Priefer U, Pühler A. A Broad Host Range Mobilization System for In Vivo Genetic Engineering: Transposon Mutagenesis in Gram Negative Bacteria. Bio/Technology. 1983;1(9):784-91.

2. Qiu X, Gurkar AU, Lory S. Interstrain transfer of the large pathogenicity island (PAPI-1) of Pseudomonas aeruginosa. Proc Natl Acad Sci U S A. 2006;103(52):19830-5.

3. Liberati NT, Urbach JM, Miyata S, Lee DG, Drenkard E, Wu G et al. An ordered, nonredundant library of Pseudomonas aeruginosa strain PA14 transposon insertion mutants. Proc Natl Acad Sci U S A. 2006;103(8):2833-8.

4. Rietsch A, Vallet-Gely I, Dove SL, Mekalanos JJ. ExsE, a secreted regulator of type III secretion genes in Pseudomonas aeruginosa. Proc Natl Acad Sci U S A. 2005;102(22):8006-11.
