# Supplementary material for: Conjugative type IVb pilus recognizes lipopolysaccharide of recipient cells to initiate PAPI-1 pathogenicity island transfer in Pseudomonas aeruginosa
Source: BMC Microbiol. 2017 Feb 7;17:31. doi: 10.1186/s12866-017-0943-4 (PMC5297154; doi:10.1186/s12866-017-0943-4)
Supplement: Additional file 2: Table S4. — Primers used in this study. (DOC 45 kb) [file 12866_2017_943_MOESM2_ESM.doc]

Table S4. Primers used in this study

| **Primer** | **Sequence (5’-3’)** | **Description** | **Source** |
| --- | --- | --- | --- |
| TnC2-US-F | GGTACCGGCAACACATTTCTCCCTCG | Amplify a fragment of 532 bp upstream of PA14_59200 gene | This study |
| TnC2-US-R | TCTAGATTGAGCCAGCCAGTTGTAGA |  |  |
| TnC2-DS-F | TCTAGACGGCTGAGAGACATCAAGGA | Amplify a fragment of 594 bp downstream of PA14_59200 gene | This study |
| TnC2-DS-R | AAGCTTGTTCAGGTTCGTCGCTATGG |  |  |
| Tc-F | TCTAGATCAGGTCGAGGTGGCCC | Amplify Tet gene from mini-CTX2 plasmid | This study |
| Tc-R | TCTAGAAGAGCGCTTTTGAAGCTAATTCGCTG |  |  |
| TnC2-Li-F | CTTGACGAGTTTGCTGCACT | Check the insert of Tet gene on the left junction | This study |
| TnC2-Li-R | GAGAAGCAGGCCATTATCGC |  |  |
| TnC2-Ri-F | GAACGGGTGCGCATAGAAAT | Check the insert of Tet gene on the right junction | This study |
| TnC2-Ri-R | TTCGACCAAGGAGCTGAACT |  |  |
| pilV2-F | ATAGGATCCCTGTCCTGCCAAAACGGG | Amplify C-terminal region of pilV2 gene (97 amino acid) | This study |
| pilV2-R | ATATGAATTCCTAGTTCACGCAGGTAACGG |  |  |
| intF | AGCTACATCGAGGCCGACTA | Check the insertion of PAPI-1 on the left junction of attL site | [1] |
| 4542F | GTGGTGATGACCTCCAACCT |  | [1] |
| sojR | CGAGCACAGAAATGTCCTGA | Check the insertion of PAPI-1 on the right junction of attR site | [1] |
| 4541F | GACAAGACCAGCCACAACCT |  |  |

References

1. Qiu X, Gurkar AU, Lory S. Interstrain transfer of the large pathogenicity island (PAPI-1) of Pseudomonas aeruginosa. Proc Natl Acad Sci U S A. 2006;103(52):19830-5.
