# Supplementary material for: Conjugative type IVb pilus recognizes lipopolysaccharide of recipient cells to initiate PAPI-1 pathogenicity island transfer in Pseudomonas aeruginosa
Source: BMC Microbiol. 2017 Feb 7;17:31. doi: 10.1186/s12866-017-0943-4 (PMC5297154; doi:10.1186/s12866-017-0943-4)
Supplement: Additional file 3: Table S2. — List of PAO1 mutants for lipopolysaccharide biosynthesis. (DOC 67 kb) [file 12866_2017_943_MOESM3_ESM.doc]

Table S2. List of PAO1 mutants for lipopolysaccharide biosynthesis

| **Number** | **PA ORF** | **Gene Abbrev.** | **Putative ORF Function** | **Position in PAO1 transposon mutant library (source: [1])** |
| --- | --- | --- | --- | --- |
| 1 | PA0705 | *migA* | alpha-1,6-rhamnosyltransferase MigA | phoAwp01q4A03 |
| 2 | PA0936 | *lpxO2* | lipopolysaccharide biosynthetic protein LpxO2 | lacZwp03q3H09 |
| 3 | PA3141 | *wbpM* | nucleotide sugar epimerase/dehydratase | (*) |
| 4 | PA3157 |  | probable acetyltransferase | phoAwp08q3G06 |
| 5 | PA3160 | *wzz* | O-antigen chain length regulator | phoAbp02q3G06 |
| 6 | PA3193 | *glk* | Glucokinase | phoAwp07q4C11 |
| 7 | PA3337 | *rfaD* | ADP-L-glycero-D-mannoheptose 6-epimerase | phoAwp05q3A01 |
| 8 | PA3552 | *arnB* | ArnB | phoAwp08q4G12 |
| 9 | PA3554 | *arnA* | ArnA | lacZwp07q3F04 |
| 10 | PA3555 | *arnD* | ArnD | phoAwp04q2C06 |
| 11 | PA3556 | *arnT* | inner membrane L-Ara4N transferase ArnT | lacZwp07q1F11 |
| 12 | PA4458 |  | conserved hypothetical protein | phoAwp07q2G01 |
| 13 | PA4512 | *lpxO1* | lipopolysaccharide biosynthetic protein LpxO1 | phoAwp07q3E07 |
| 14 | PA4661 | *pagL* | Lipid A 3-O-deacylase | phoAbp02q4E08 |
| 15 | PA5001 |  | hypothetical protein | phoAwp01q3H11 |
| 16 | PA5002 |  | hypothetical protein | lacZbp03q3E06 |
| 17 | PA5005 |  | probable carbamoyl transferase | phoAwp09q3B06 |
| 18 | PA5009 | *waaP* | lipopolysaccharide kinase WaaP | phoAwp05q4G09 |
| 19 | PA5011 | *waaC* | heptosyltransferase I | lacZwp04q4G06 |
| 20 | PA5012 | *waaF* | heptosyltransferase II | lacZwp08q1C03 |
| 21 | PA5447 | *wbpZ* | glycosyltransferase WbpZ | lacZwp02q1H10 |
| 22 | PA5448 | *wbpY* | glycosyltransferase WbpY | phoAwp02q1F12 |
| 23 | PA5449 | *wbpX* | glycosyltransferase WbpX | lacZwp01q4A02 |
| 24 | PA5450 | *wzt* | ABC subunit of A-band LPS efflux transporter | phoAwp10q1E09 |
| 25 | PA5452 | *wbpW* | phosphomannose isomerase/GDP-mannose WbpW | lacZwp08q4H11 |
| 26 | PA5453 | *gmd* | GDP-mannose 4,6-dehydratase | lacZwp02q3E02 |
| 27 | PA5454 | *rmd* | oxidoreductase Rmd | lacZwp01q1B08 |
| 28 | PA5455 |  | hypothetical protein | phoAwp08q4H06 |
| 29 | PA5456 |  | hypothetical protein | lacZwp02q4C05 |
| 30 | PA5457 |  | hypothetical protein | lacZwp06q1F08 |
| 31 | PA5458 |  | hypothetical protein | phoAwp10q1C10 |
| 32 | PA5459 |  | hypothetical protein | phoAwp08q1B12 |
| 33 | PA5322 | *algC* | phosphomannomutase | phoAwp07q4D07 |

(*): Lory’s lab collection

References

1. Jacobs MA, Alwood A, Thaipisuttikul I, Spencer D, Haugen E, Ernst S et al. Comprehensive transposon mutant library of Pseudomonas aeruginosa. Proc Natl Acad Sci U S A. 2003;100(24):14339-44.
