# Supplementary material for: Conjugative type IVb pilus recognizes lipopolysaccharide of recipient cells to initiate PAPI-1 pathogenicity island transfer in Pseudomonas aeruginosa
Source: BMC Microbiol. 2017 Feb 7;17:31. doi: 10.1186/s12866-017-0943-4 (PMC5297154; doi:10.1186/s12866-017-0943-4)
Supplement: Additional file 4: Table S3. — Genes involved or potentially involved in Common Polysaccharide Antigen (A-band LPS) biosynthesis. This table was reproduced by King et al., 2009. (DOC 69 kb) [file 12866_2017_943_MOESM4_ESM.doc]

Table S3. Genes involved or potentially involved in Common Polysaccharide Antigen (A-band LPS) biosynthesis. This table was reproduced by King *et al.*, 2009 [1].

| **Gene** | **Related proteins (% identity)** | **Proposed/demonstrated function** | **Key reference** |
| --- | --- | --- | --- |
| *wbpZ/PA5447* | 52% *E. coli* O9a WbdC | Glycosyltransferase (GT-4) | [2] |
| *wbpY/PA5448* | 34% *E. coli* O9a WbdB | Glycosyltransferase (GT-4) | [2] |
| *wbpX/PA5449* | 33% *E. coli* O9a WbdA C-terminal domain (over 301 amino acids) | Glycosyltransferase (GT-4) | [2] |
|  | 25% *E. coli* O9a WbdA N-terminal domain (over 262 amino acids) |  |  |
| *wzt/PA5450* | 61% *E. coli* O8 Wzt | ABC transporter | [3] |
| *wzm/PA5451* | 56% *E. coli* O8 Wzm | ABC transporter | [3] |
| *wbpW/PA5452* | 46% *P. aeruginosa* AlgA | D-Man-6-phosphate isomerase / GDP-D-Man pyrophosphorylase | [4] |
|  | 60% *P. aeruginosa* PslB |  |  |
| *gmd/PA5453* | 47% *E. coli* GMD | GDP-D-Man 4,6-dehydratase | [5] |
| *rmd/PA5454* | 33% *Aneurinibacillus thermoaerophilus* RMD | GDP-D-Rha synthase | [5] |
| *PA5455* |  | Glycosyltransferase (GT-4) |  |
| *PA5456* |  | Glycosyltransferase (GT-4) |  |
| *PA5457* | 20% *E. coli* O8 WbdD (over 149 amino acids) | Methyltransferase |  |
| *PA5458* | 24% *Staphylococcus aureus* OatA | Acetyltransferase |  |
| *PA5459* | 23% *E. coli* O8 WbdD (over 139 amino acids) | Methyltransferase |  |
| *algC/PA5322* | 31% *E. coli* ManB | Phosphomannomutase/phosphoglucomutase | [6] |

The classification of Glycosyltransferase (GT) family is provided by the CAZy database [7].

**References**

1. King JD, Kocincova D, Westman EL, Lam JS. Review: Lipopolysaccharide biosynthesis in Pseudomonas aeruginosa. Innate Immun. 2009;15(5):261-312.

2. Rocchetta HL, Burrows LL, Pacan JC, Lam JS. Three rhamnosyltransferases responsible for assembly of the A-band D-rhamnan polysaccharide in Pseudomonas aeruginosa: a fourth transferase, WbpL, is required for the initiation of both A-band and B-band lipopolysaccharide synthesis. Mol Microbiol. 1998;28(6):1103-19.

3. Rocchetta HL, Lam JS. Identification and functional characterization of an ABC transport system involved in polysaccharide export of A-band lipopolysaccharide in Pseudomonas aeruginosa. J Bacteriol. 1997;179(15):4713-24.

4. Rocchetta HL, Pacan JC, Lam JS. Synthesis of the A-band polysaccharide sugar D-rhamnose requires Rmd and WbpW: identification of multiple AlgA homologues, WbpW and ORF488, in Pseudomonas aeruginosa. Mol Microbiol. 1998;29(6):1419-34.

5. King JD, Poon KK, Webb NA, Anderson EM, McNally DJ, Brisson JR et al. The structural basis for catalytic function of GMD and RMD, two closely related enzymes from the GDP-D-rhamnose biosynthesis pathway. FEBS J. 2009;276(10):2686-700.

6. Zielinski NA, Chakrabarty AM, Berry A. Characterization and regulation of the Pseudomonas aeruginosa algC gene encoding phosphomannomutase. J Biol Chem. 1991;266(15):9754-63.

7. Cantarel BL, Coutinho PM, Rancurel C, Bernard T, Lombard V, Henrissat B. The Carbohydrate-Active EnZymes database (CAZy): an expert resource for Glycogenomics. Nucleic Acids Res. 2009;37:D233-8.
