# Supplementary figures and images for: Conjugative type IVb pilus recognizes lipopolysaccharide of recipient cells to initiate PAPI-1 pathogenicity island transfer in Pseudomonas aeruginosa
Source: BMC Microbiol. 2017 Feb 7;17:31. doi: 10.1186/s12866-017-0943-4 (PMC5297154; doi:10.1186/s12866-017-0943-4)

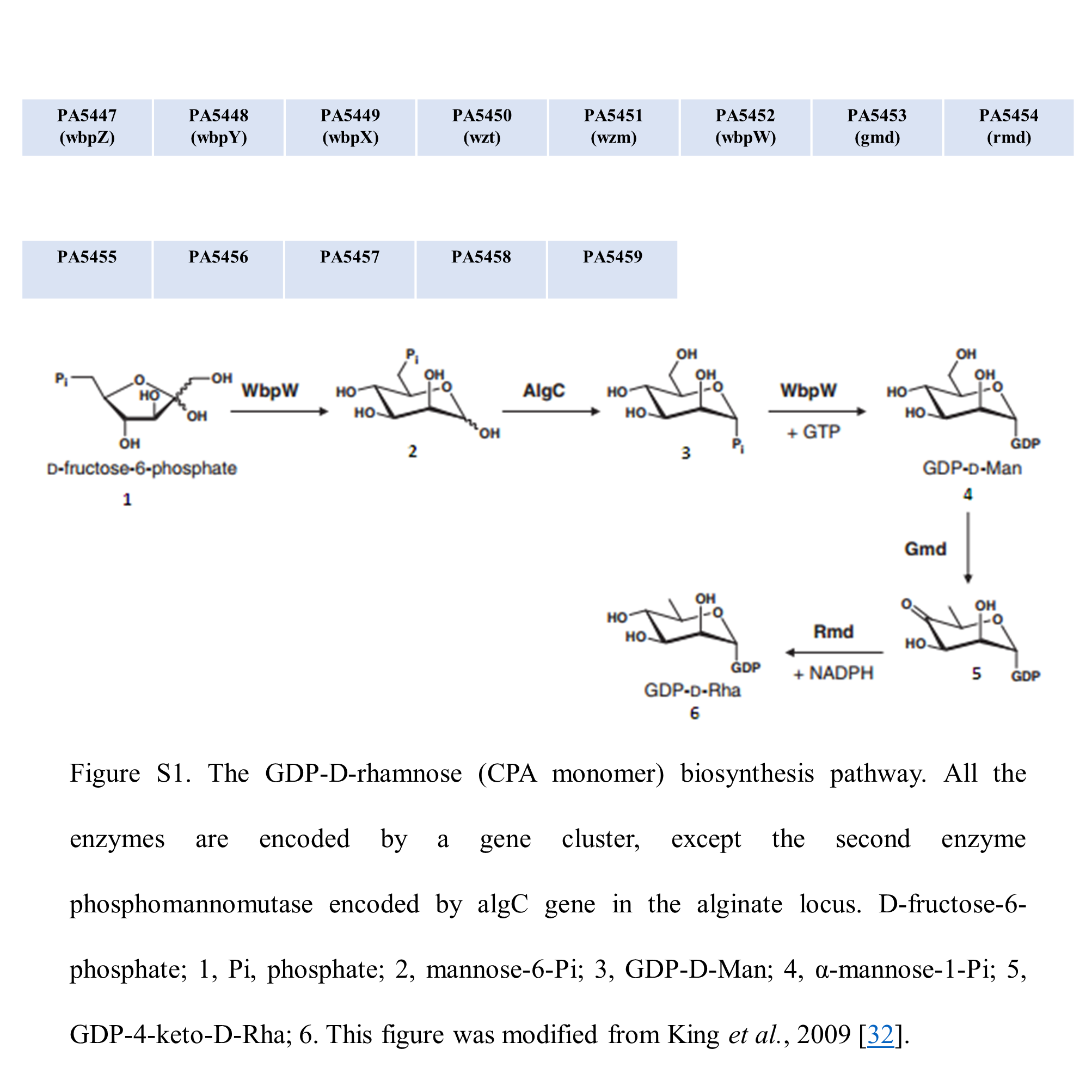

Supplement: Additional file 5: Figure S1. — The GDP-D-rhamnose (CPA monomer) biosynthesis pathway. All the enzymes are encoded by a gene cluster, except the second enzyme phosphomannomutase encoded by algC gene in the alginate locus. D-fructose-6-phosphate; 1, Pi, phosphate; 2, mannose-6-Pi; 3, GDP-D-Man; 4, α-mannose-1-Pi; 5, GDP-4-keto-D-Rha; 6. This figure was modified from King et al., 2009 [32]. (TIF 572 kb) [file 12866_2017_943_MOESM5_ESM.tif]

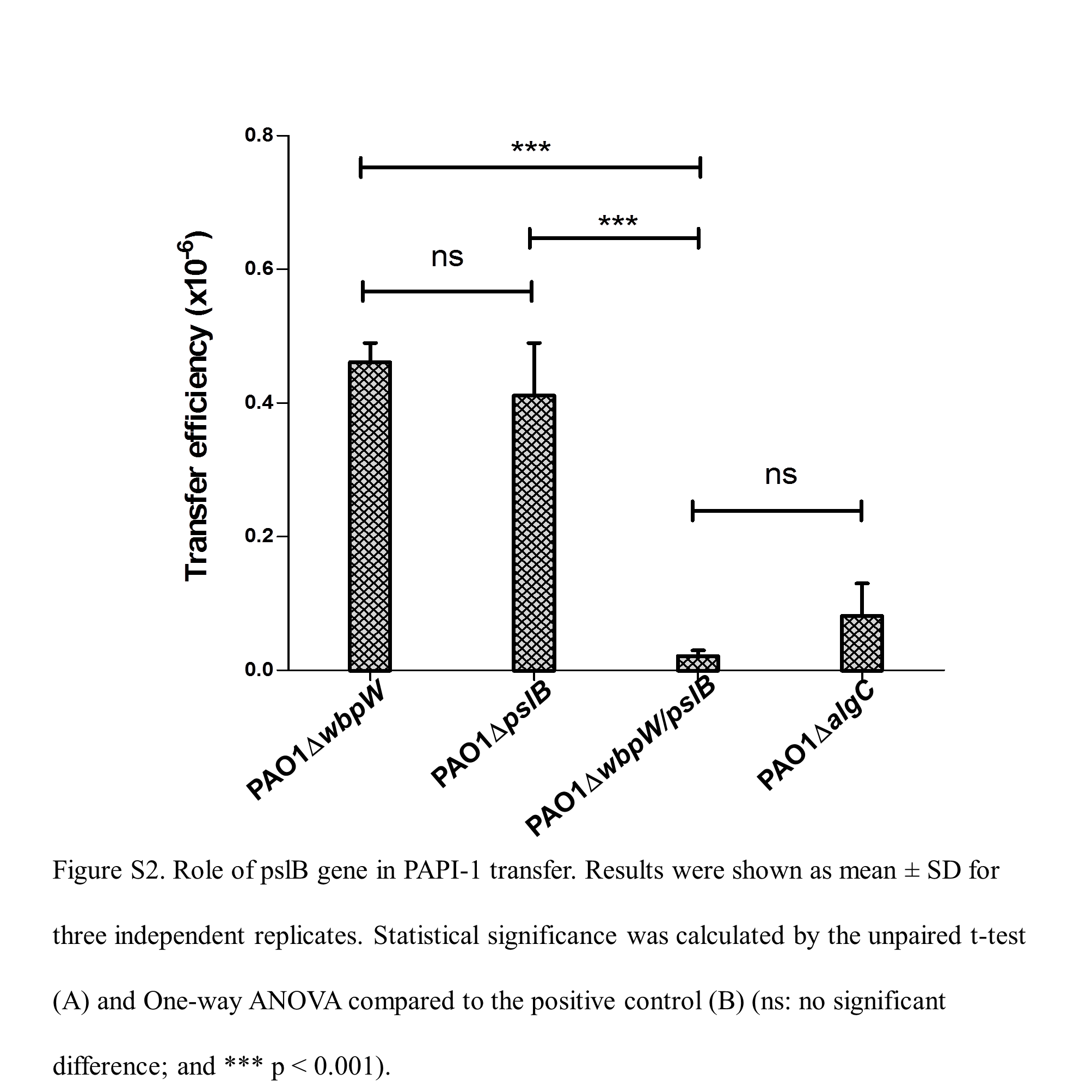

Supplement: Additional file 6: Figure S2. — Role of pslB gene in PAPI-1 transfer. Results were shown as mean ± SD for three independent replicates. Statistical significance was analyzed by One-way ANOVA compared to the positive control (ns: no significant difference; and *** p < 0.001). (TIF 217 kb) [file 12866_2017_943_MOESM6_ESM.tif]

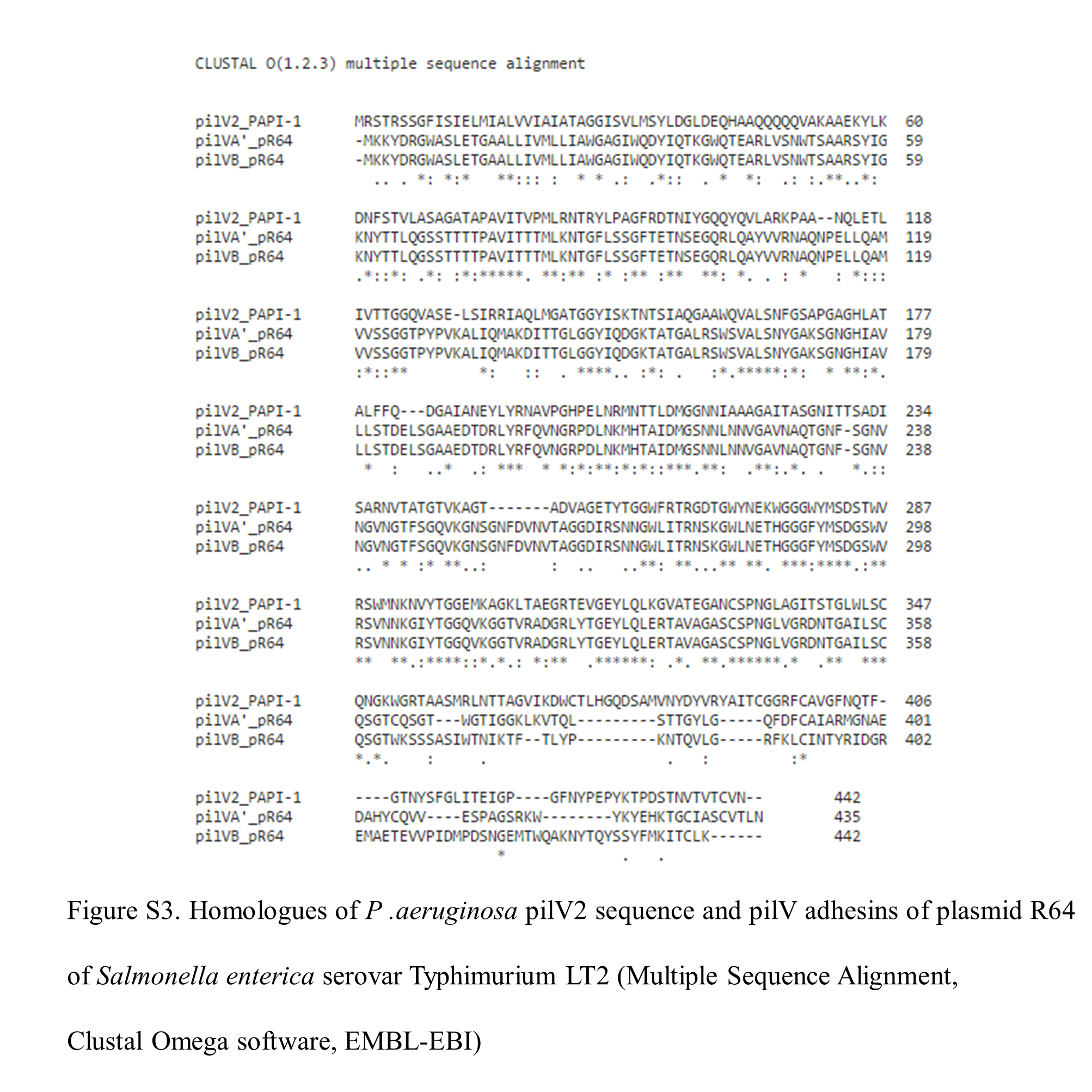

Supplement: Additional file 11: Figure S3. — Homologues of P.aeruginosa pilV2 sequence and pilV adhesins of plasmid R64 of Salmonella enterica serovar Typhimurium LT2 (Multiple Sequence Alignment, Clustal Omega software, EMBL-EBI). (TIF 2355 kb) [file 12866_2017_943_MOESM11_ESM.tif]
