# Supplementary material for: Conjugative type IVb pilus recognizes lipopolysaccharide of recipient cells to initiate PAPI-1 pathogenicity island transfer in Pseudomonas aeruginosa
Source: BMC Microbiol. 2017 Feb 7;17:31. doi: 10.1186/s12866-017-0943-4 (PMC5297154; doi:10.1186/s12866-017-0943-4)
Supplement: Additional file 7: Table S5. — Transfer efficiency of different strains and mutants. (DOCX 19 kb) [file 12866_2017_943_MOESM7_ESM.docx]

**Table S5. Transfer efficiency of different strains and mutants**

| **Mutants** | **Transfer efficiency (10^-6^)** | | | **Transfer efficiency (10^-6^)** | |
| --- | --- | --- | --- | --- | --- |
|  | **Rep 1** | **Rep 2** | **Rep 3** | **Mean** | **SD** |
| PA0705 | 1.929 | 1.583 | 2.327 | 1.946 | 0.372 |
| PA0936 | 2.212 | 2.418 | 1.841 | 2.157 | 0.292 |
| PA3141 | 1.747 | 1.959 | 2.196 | 1.967 | 0.225 |
| PA3157 | 0.865 | 0.715 | 1.030 | 0.870 | 0.157 |
| PA3160 | 1.844 | 2.449 | 1.949 | 2.081 | 0.323 |
| PA3193 | 1.957 | 2.114 | 1.342 | 1.804 | 0.408 |
| PA3337 | 0.844 | 0.867 | 0.607 | 0.773 | 0.144 |
| PA3552 | 1.731 | 2.308 | 2.064 | 2.034 | 0.290 |
| PA3554 | 1.016 | 1.158 | 1.063 | 1.079 | 0.073 |
| PA3555 | 1.106 | 1.219 | 1.842 | 1.389 | 0.396 |
| PA3556 | 0.905 | 0.556 | 0.563 | 0.674 | 0.200 |
| PA4458 | 1.056 | 1.226 | 0.985 | 1.089 | 0.124 |
| PA4512 | 0.864 | 0.470 | 0.750 | 0.694 | 0.203 |
| PA4661 | 1.930 | 2.024 | 1.720 | 1.891 | 0.156 |
| PA5001 | 0.101 | 0.037 | 0.036 | 0.058 | 0.037 |
| PA5002 | 0.952 | 1.009 | 0.988 | 0.983 | 0.029 |
| PA5005 | 0.792 | 0.750 | 0.737 | 0.760 | 0.029 |
| PA5009 | 1.342 | 0.944 | 1.517 | 1.268 | 0.293 |
| PA5011 | 1.877 | 2.287 | 1.809 | 1.991 | 0.258 |
| PA5012 | 1.023 | 1.455 | 1.523 | 1.333 | 0.271 |
| PA5447 | 0.000 | 0.000 | 0.000 | 0.000 | 0.000 |
| PA5448 | 0.081 | 0.052 | 0.021 | 0.051 | 0.030 |
| PA5449 | 0.000 | 0.000 | 0.000 | 0.000 | 0.000 |
| PA5450 | 0.000 | 0.000 | 0.000 | 0.000 | 0.000 |
| PA5452 | 1.679 | 1.033 | 0.829 | 1.180 | 0.443 |
| PA5453 | 0.017 | 0.056 | 0.026 | 0.033 | 0.020 |
| PA5454 | 0.418 | 0.188 | 0.144 | 0.250 | 0.147 |
| PA5455 | 0.102 | 0.056 | 0.071 | 0.076 | 0.024 |
| PA5456 | 0.292 | 0.029 | 0.036 | 0.119 | 0.150 |
| PA5457 | 2.513 | 2.986 | 2.206 | 2.568 | 0.393 |
| PA5458 | 1.766 | 1.034 | 1.463 | 1.421 | 0.368 |
| PA5459 | 0.038 | 0.072 | 0.018 | 0.043 | 0.027 |
| PAO1Δ*WbpW* | 0.484 | 0.424 | 0.481 | 0.463 | 0.034 |
| PAO1Δ*pslB* | 0.481 | 0.368 | 0.343 | 0.397 | 0.073 |
| PAO1Δ*WbpW/pslB* | 0.031 | 0.014 | 0.018 | 0.021 | 0.009 |
| PA5322 (NEG) | 0.000 | 0.018 | 0.054 | 0.024 | 0.028 |
| PAO1 (POS) | 1.179 | 1.420 | 1.662 | 1.420 | 0.242 |
