# Supplementary material for: Conjugative type IVb pilus recognizes lipopolysaccharide of recipient cells to initiate PAPI-1 pathogenicity island transfer in Pseudomonas aeruginosa
Source: BMC Microbiol. 2017 Feb 7;17:31. doi: 10.1186/s12866-017-0943-4 (PMC5297154; doi:10.1186/s12866-017-0943-4)
Supplement: Additional file 8: Table S7. — PAPI-1 transfer inhibition following addition of OMs. (DOCX 24 kb) [file 12866_2017_943_MOESM8_ESM.docx]

**Table S7. PAPI-1 transfer inhibition following addition of OMs**

| **Strains/Mutants** | **OMs addition (µg)** | **Transfer efficiency**  **(10^-6^)** | | | **Transfer inhibition index (%)** | | | **Transfer inhibition index (%)** | |
| --- | --- | --- | --- | --- | --- | --- | --- | --- | --- |
|  |  | **Rep 1** | **Rep 2** | **Rep 3** | **Rep 1** | **Rep 2** | **Rep 3** | **Mean** | **SD** |
| PAO1Δ*WbpM*  (+A,-B) | 0.5 | 1.46 | 1.70 | 1.53 | 85.41 | 86.63 | 83.13 | 85.06 | 1.77 |
|  | 1 | 1.05 | 1.33 | 1.14 | 61.85 | 68.09 | 62.03 | 63.99 | 3.55 |
|  | 2 | 0.72 | 0.92 | 0.70 | 42.06 | 46.86 | 38.05 | 42.32 | 4.41 |
|  | 5 | 0.60 | 0.72 | 0.61 | 35.20 | 36.53 | 33.48 | 35.07 | 1.53 |
|  | 10 | 0.39 | 0.52 | 0.35 | 23.05 | 26.72 | 19.33 | 23.03 | 3.69 |
|  | 15 | 0.34 | 0.43 | 0.32 | 19.70 | 22.15 | 17.69 | 19.85 | 2.23 |
| PAO1Δ*wzx* (+A,-B) | 0.5 | 1.32 | 1.58 | 1.34 | 77.38 | 80.48 | 73.18 | 77.01 | 3.66 |
|  | 1 | 0.85 | 1.04 | 0.88 | 49.58 | 53.19 | 48.03 | 50.27 | 2.65 |
|  | 2 | 0.67 | 0.79 | 0.65 | 39.11 | 40.48 | 35.59 | 38.39 | 2.52 |
|  | 5 | 0.34 | 0.39 | 0.27 | 19.77 | 20.09 | 14.49 | 18.12 | 3.15 |
|  | 10 | 0.24 | 0.29 | 0.25 | 14.16 | 14.85 | 13.86 | 14.29 | 0.51 |
|  | 15 | 0.22 | 0.30 | 0.20 | 12.88 | 15.57 | 10.64 | 13.03 | 2.47 |
| PAO1Δ*rmd* (-A,+B) | 0.5 | 1.61 | 1.85 | 1.71 | 94.59 | 94.31 | 92.99 | 93.96 | 0.85 |
|  | 1 | 1.56 | 1.80 | 1.66 | 91.76 | 91.91 | 90.55 | 91.41 | 0.75 |
|  | 2 | 1.52 | 1.85 | 1.67 | 89.40 | 94.62 | 90.80 | 91.60 | 2.70 |
|  | 5 | 1.50 | 1.68 | 1.55 | 88.00 | 85.88 | 84.19 | 86.02 | 1.91 |
|  | 10 | 1.59 | 1.69 | 1.61 | 93.40 | 86.50 | 87.55 | 89.15 | 3.72 |
|  | 15 | 1.53 | 1.74 | 1.65 | 89.99 | 89.03 | 90.10 | 89.71 | 0.58 |
| PAO1Δ*algC* (-A,+B) | 0.5 | 1.57 | 1.87 | 1.66 | 92.13 | 95.47 | 90.63 | 92.74 | 2.48 |
|  | 1 | 1.48 | 1.75 | 1.55 | 86.63 | 89.59 | 84.52 | 86.91 | 2.54 |
|  | 2 | 1.52 | 1.80 | 1.60 | 89.38 | 92.02 | 87.26 | 89.55 | 2.39 |
|  | 5 | 1.51 | 1.66 | 1.51 | 88.49 | 84.95 | 82.43 | 85.29 | 3.04 |
|  | 10 | 1.37 | 1.62 | 1.45 | 80.18 | 82.74 | 79.21 | 80.71 | 1.83 |
|  | 15 | 1.43 | 1.72 | 1.51 | 84.13 | 87.68 | 82.09 | 84.63 | 2.83 |
| PAO1- | 0.5 | 1.44 | 1.75 | 1.46 | 84.66 | 89.15 | 79.65 | 84.49 | 4.75 |
|  | 1 | 0.84 | 1.03 | 0.90 | 49.40 | 52.57 | 49.14 | 50.37 | 1.91 |
|  | 2 | 0.54 | 0.60 | 0.53 | 31.70 | 30.44 | 28.89 | 30.34 | 1.41 |
|  | 5 | 0.34 | 0.40 | 0.29 | 19.97 | 20.57 | 16.05 | 18.86 | 2.45 |
|  | 10 | 0.27 | 0.34 | 0.26 | 15.71 | 17.51 | 14.42 | 15.88 | 1.55 |
|  | 15 | 0.21 | 0.31 | 0.17 | 12.28 | 15.65 | 9.33 | 12.42 | 3.16 |
| PAO1+ | 0.5 | 1.64 | 1.90 | 1.73 | 96.00 | 97.15 | 94.20 | 95.78 | 1.48 |
|  | 1 | 1.46 | 1.74 | 1.54 | 85.45 | 88.67 | 84.09 | 86.07 | 2.35 |
|  | 2 | 1.56 | 1.83 | 1.62 | 91.67 | 93.27 | 88.20 | 91.05 | 2.59 |
|  | 5 | 1.44 | 1.75 | 1.59 | 84.33 | 89.36 | 86.44 | 86.71 | 2.53 |
|  | 10 | 1.42 | 1.67 | 1.49 | 83.03 | 85.11 | 81.35 | 83.16 | 1.88 |
|  | 15 | 1.39 | 1.64 | 1.55 | 81.48 | 83.56 | 84.23 | 83.09 | 1.43 |
| PA14+ | 0.5 | 1.56 | 1.86 | 1.69 | 91.35 | 94.75 | 92.07 | 92.72 | 1.79 |
|  | 1 | 1.35 | 1.49 | 1.35 | 79.20 | 76.19 | 73.48 | 76.29 | 2.86 |
|  | 2 | 0.97 | 1.16 | 0.99 | 56.75 | 59.16 | 53.91 | 56.61 | 2.63 |
|  | 5 | 0.61 | 0.75 | 0.61 | 35.55 | 38.40 | 33.38 | 35.78 | 2.52 |
|  | 10 | 0.67 | 0.81 | 0.70 | 39.11 | 41.22 | 38.40 | 39.58 | 1.47 |
|  | 15 | 0.52 | 0.66 | 0.53 | 30.42 | 33.48 | 28.79 | 30.90 | 2.38 |
| PA14- | 0.5 | 1.54 | 1.72 | 1.59 | 90.22 | 87.61 | 86.55 | 88.13 | 1.89 |
|  | 1 | 1.55 | 1.81 | 1.65 | 90.67 | 92.20 | 89.83 | 90.90 | 1.20 |
|  | 2 | 1.60 | 1.85 | 1.73 | 93.64 | 94.47 | 94.29 | 94.13 | 0.43 |
|  | 5 | 1.48 | 1.74 | 1.62 | 86.58 | 88.69 | 88.42 | 87.90 | 1.15 |
|  | 10 | 1.48 | 1.66 | 1.58 | 87.02 | 84.72 | 86.13 | 85.96 | 1.16 |
|  | 15 | 1.46 | 1.72 | 1.57 | 85.63 | 87.70 | 85.41 | 86.25 | 1.26 |
| Control (PA14Δ*TnC2* and PAO1) | 0 | 1.70 | 1.96 | 1.84 | 100.00 | 100.00 | 100.00 | 100.00 | 0.00 |
