# Supplementary material for: Conjugative type IVb pilus recognizes lipopolysaccharide of recipient cells to initiate PAPI-1 pathogenicity island transfer in Pseudomonas aeruginosa
Source: BMC Microbiol. 2017 Feb 7;17:31. doi: 10.1186/s12866-017-0943-4 (PMC5297154; doi:10.1186/s12866-017-0943-4)
Supplement: Additional file 9:Table S8. — PAPI-1 transfer inhibition following addition of LPSs. (DOCX 22 kb) [file 12866_2017_943_MOESM9_ESM.docx]

**Table S8. PAPI-1 transfer inhibition following addition of LPSs**

| **Strains/Mutants** | **LPSs addition (µg)** | **Transfer efficiency (10^-6^)** | | | **Transfer inhibition index (%)** | | | **Transfer inhibition index (%)** | |
| --- | --- | --- | --- | --- | --- | --- | --- | --- | --- |
|  |  | **Rep 1** | **Rep 2** | **Rep 3** | **Rep 1** | **Rep 2** | **Rep 3** | **Mean** | **SD** |
| PAO1Δ*WbpM* (+A,-B) | 1 | 0.83 | 0.84 | 0.77 | 49.14 | 64.67 | 59.72 | 57.84 | 7.93 |
|  | 5 | 0.28 | 0.48 | 0.31 | 16.70 | 37.28 | 23.91 | 25.96 | 10.44 |
|  | 10 | 0.36 | 0.29 | 0.33 | 21.46 | 22.62 | 25.95 | 23.35 | 2.33 |
|  | 15 | 0.17 | 0.14 | 0.19 | 9.86 | 10.52 | 14.53 | 11.64 | 2.53 |
| PAO1Δ*wzx*  (+A,-B) | 1 | 1.29 | 0.90 | 0.94 | 76.51 | 69.76 | 72.87 | 73.05 | 3.38 |
|  | 5 | 0.49 | 0.56 | 0.48 | 29.22 | 43.32 | 37.60 | 36.71 | 7.09 |
|  | 10 | 0.49 | 0.43 | 0.49 | 29.20 | 33.45 | 38.49 | 33.71 | 4.65 |
|  | 15 | 0.18 | 0.18 | 0.23 | 10.67 | 13.94 | 18.25 | 14.28 | 3.80 |
| PAO1Δ*rmd*  (-A,+B) | 1 | 1.59 | 1.13 | 1.28 | 94.19 | 87.46 | 99.91 | 93.85 | 6.23 |
|  | 5 | 1.48 | 1.28 | 1.31 | 87.66 | 99.00 | 101.99 | 96.21 | 7.56 |
|  | 10 | 1.39 | 1.27 | 1.18 | 82.10 | 98.65 | 91.87 | 90.87 | 8.32 |
|  | 15 | 1.44 | 1.09 | 1.28 | 85.15 | 84.31 | 99.40 | 89.62 | 8.48 |
| PAO1Δa*lgC*  (-A,+B) | 1 | 1.44 | 1.05 | 1.12 | 85.12 | 81.11 | 87.12 | 84.45 | 3.06 |
|  | 5 | 1.45 | 1.16 | 1.03 | 85.74 | 89.91 | 80.36 | 85.34 | 4.78 |
|  | 10 | 1.52 | 1.08 | 1.19 | 89.78 | 83.87 | 92.82 | 88.82 | 4.55 |
|  | 15 | 1.37 | 0.88 | 1.09 | 80.79 | 68.42 | 85.19 | 78.13 | 8.70 |
| PAO1 - | 1 | 0.92 | 0.72 | 0.88 | 54.37 | 55.36 | 68.84 | 59.52 | 8.08 |
|  | 5 | 0.36 | 0.49 | 0.46 | 21.48 | 37.55 | 36.03 | 31.69 | 8.87 |
|  | 10 | 0.37 | 0.31 | 0.38 | 21.82 | 24.36 | 29.66 | 25.28 | 4.00 |
|  | 15 | 0.36 | 0.13 | 0.15 | 21.20 | 10.01 | 11.38 | 14.20 | 6.10 |
| PAO1+ | 1 | 1.55 | 0.97 | 1.22 | 91.76 | 75.29 | 94.80 | 87.28 | 10.49 |
|  | 5 | 1.62 | 1.16 | 1.15 | 96.04 | 89.55 | 89.68 | 91.75 | 3.71 |
|  | 10 | 1.33 | 1.08 | 1.01 | 78.90 | 83.23 | 78.51 | 80.21 | 2.62 |
|  | 15 | 1.46 | 1.07 | 1.07 | 86.17 | 82.80 | 83.34 | 84.10 | 1.81 |
| PA14+ | 1 | 1.45 | 0.95 | 0.89 | 85.70 | 73.21 | 69.30 | 76.07 | 8.56 |
|  | 5 | 1.24 | 0.98 | 1.11 | 73.26 | 75.76 | 86.37 | 78.46 | 6.96 |
|  | 10 | 1.16 | 1.04 | 0.93 | 68.49 | 80.60 | 72.38 | 73.82 | 6.19 |
|  | 15 | 1.22 | 0.95 | 1.16 | 72.32 | 73.60 | 90.38 | 78.77 | 10.07 |
| PA14- | 1 | 1.33 | 0.85 | 1.19 | 78.43 | 65.60 | 92.68 | 78.90 | 13.55 |
|  | 5 | 0.97 | 0.91 | 0.92 | 57.61 | 70.13 | 71.83 | 66.52 | 7.76 |
|  | 10 | 1.54 | 1.02 | 0.84 | 91.28 | 78.87 | 65.59 | 78.58 | 12.85 |
|  | 15 | 1.19 | 1.11 | 0.97 | 70.65 | 85.89 | 75.48 | 77.34 | 7.79 |
| *S. enterica* | 1 | 1.42 | 1.20 | 1.15 | 83.73 | 92.67 | 89.53 | 88.64 | 4.53 |
|  | 5 | 1.47 | 1.09 | 1.15 | 87.24 | 84.51 | 89.41 | 87.05 | 2.46 |
|  | 10 | 1.36 | 1.11 | 1.08 | 80.62 | 86.25 | 84.25 | 83.71 | 2.85 |
|  | 15 | 1.67 | 1.12 | 0.98 | 98.62 | 86.60 | 76.46 | 87.23 | 11.09 |
| Control (PA14Δ*TnC2* and PAO1) | 0 | 1.69 | 1.29 | 1.28 | 100.00 | 100.00 | 100.00 | 100.00 | 0.00 |
