# Supplementary material for: Conjugative type IVb pilus recognizes lipopolysaccharide of recipient cells to initiate PAPI-1 pathogenicity island transfer in Pseudomonas aeruginosa
Source: BMC Microbiol. 2017 Feb 7;17:31. doi: 10.1186/s12866-017-0943-4 (PMC5297154; doi:10.1186/s12866-017-0943-4)
Supplement: Additional file 10: Table S6. — Transfer efficiency of different combinations of donors and recipients with or without PAPI-1. (DOCX 14 kb) [file 12866_2017_943_MOESM10_ESM.docx]

Table S6. Transfer efficiency of different combinations of donors and recipients with or without PAPI-1

| **Donor** | **Recipients** | **Transfer efficiency** | | | **Transfer efficiency** | |
| --- | --- | --- | --- | --- | --- | --- |
|  |  | **Rep 1** | **Rep 2** | **Rep 3** | **Mean** | **SD** |
| PAO1+ | PAO1 + | 5.13E-09 | 1.02E-08 | 9.11E-09 | 8.12E-09 | 2.65E-09 |
|  | PAO1 - | 2.61E-06 | 1.34E-06 | 3.34E-06 | 2.42E-06 | 1.01E-06 |
| PA14+ | PAO1 + | 3.6E-09 | 4.34E-09 | 7.78E-09 | 5.23E-09 | 2.22E-09 |
|  | PAO1 - | 2.59E-06 | 4.51E-06 | 8.79E-07 | 2.66E-06 | 1.81E-06 |
|  | PA14 + | 3.59E-09 | 1.87E-09 | 2.55E-09 | 2.67E-09 | 8.65E-10 |
|  | PA14 - | 5.10E-08 | 3.78E-09 | 9.45E-08 | 4.97E-08 | 4.53E-08 |
